# Simultaneous couch and gantry dynamic arc rotation (CG‐Darc) in the treatment of breast cancer with accelerated partial breast irradiation (APBI): a feasibility study

**DOI:** 10.1120/jacmp.v14i1.4035

**Published:** 2013-01-07

**Authors:** Carmen C. Popescu, Wayne A. Beckham, Veronica V. Patenaude, Ivo A. Olivotto, Maria T. Vlachaki

**Affiliations:** ^1^ Radiation Therapy Program British Columbia Cancer Agency‐Vancouver Island Centre Victoria British Columbia; ^2^ Physics and Astronomy Department University of Victoria Victoria British Columbia; ^3^ Division of Radiation Oncology and Developmental Radiotherapeutics University of British Columbia Vancouver British Columbia Canada

**Keywords:** breast cancer, partial breast radiation, 3D CRT, RapidArc, CG‐Darc

## Abstract

The purpose of this study was to compare the dosimetry of CG‐Darc with three‐dimensional conformal radiation therapy (3D CRT) and volumetric‐modulated arc therapy (RapidArc) in the treatment of breast cancer with APBI. CG‐Darc plans were generated using two tangential couch arcs combined with a simultaneous noncoplanar gantry arc. The dynamic couch arc was modeled by consecutive IMRT fields at 10° intervals. RapidArc plans used a single partial arc with an avoidance sector, preventing direct beam exit into the thorax. CG‐Darc and RapidArc plans were compared with 3D CRT in 20 patients previously treated with 3D CRT (group A), and in 15 additional patients who failed the dosimetric constraints of the Canadian trial and of NSABP B‐39/RTOG 0413 for APBI (group B). CG‐Darc resulted in superior target coverage compared to 3D CRT and RapidArc (V95%: 98.2% vs. 97.1% and 95.7%). For outer breast lesions, CG‐Darc and RapidArc significantly reduced the ipsilateral breast V50% by 8% in group A and 15% in group B (p<0.05) as compared with 3D CRT. For inner and centrally located lesions, CG‐Darc resulted in significant ipsilateral lung V10% reduction when compared to 3D CRT and RapidArc (10.7% vs. 12.6% and 20.7% for group A, and 15.1% vs. 25.2% and 27.3% for group B). Similar advantage was observed in the dosimetry of contralateral breast where the percent maximum dose for CG‐Darc, 3D CRT, and RapidArc were 3.9%, 6.3%, and 5.8% for group A and 4.3%, 9.2%, and 6.3% for group B, respectively (p<0.05). CG‐Darc achieved superior target coverage while decreasing normal tissue dose even in patients failing APBI dose constraints. Consequently, this technique has the potential of expanding the use of APBI to patients currently ineligible for such treatment. Modification of the RapidArc algorithm will be necessary to link couch and gantry rotation with variable dose rate and, therefore, enable the use of CG‐Darc in clinical practice.

PACS number: 80

## I. INTRODUCTION

While whole breast radiation therapy (WBRT) has been established as the standard of care in patients who have undergone lumpectomy, other techniques combining accelerated fractionation with partial breast radiotherapy fields are attractive alternative options. These techniques shorten treatment duration from several weeks to one week, while also reducing the high‐dose radiation exposure of ipsilateral breast tissue and surrounding organs at risk (OARs) such as lung, heart, and contralateral breast. Accelerated partial breast irradiation (APBI) utilizing three‐dimensional conformal radiation therapy (3D CRT) is being evaluated as an alternative to WBRT in two North American randomized trials. The Canadian Randomized Trial of Accelerated Partial Breast Irradiation (RAPID)^(^ ^1^ ^)^ completed accrual in July 2011 with over 2200 patients randomized between APBI using 3D CRT external‐beam radiation therapy or WBRT. The NSABP B‐39/RTOG 0413 trial^(^ ^2^ ^)^ is ongoing and randomizes eligible women between WBRT and APBI with either 3D CRT or brachytherapy techniques. A three‐year report from a Canadian multicenter study on APBI using 3D CRT^(^ ^3^ ^)^ showed acceptable toxicity and cosmesis with excellent control and disease‐free survival. Initial efficacy results (locoregional control and toxicity) of RTOG 0319 using 3D CRT to deliver APBI^(^ ^4^ ^)^ were comparable with other published studies with similar fallow‐up. These preliminary data support continued accrual to APBI trials, but also emphasize the need for further follow‐up to fully evaluate long‐term tumor control and late toxicity.

Using 3D CRT and the NASBP B‐39/RTOG0413 dose guidelines, Hepel et al.^(^ ^5^ ^)^ found a remarkably high rate of moderate‐to‐severe late toxicity (especially subcutaneous fibrosis) in 6 out of 60 patients treated with APBI. The medial follow‐up was 15 months and the toxicity was associated with larger volumes of irradiated ipsilateral breast tissue. Recht at el.^(^ ^6^ ^)^ observed a higher risk of pneumonitis above 15% in APBI patients treated with 3D CRT when lung volumes receiving 5, 10, and 20 Gy exceed 20%, 10%, and 3% respectively. In an attempt to minimize the dose to OARs, other treatment modalities have been used for APBI including IMRT, tomotherapy, and proton therapy. IMRT showed improved ipsilateral breast and other normal tissue dose sparing compared with 3D CRT^(^ ^7^)(^8^ ^)^ with very low acute toxicity.^(^ ^9^ ^)^ Tomotherapy also reduced the dose to ipsilateral breast, but at the cost of considerable increase in lung and heart dose.^(^ ^10^ ^)^ Protons have been proven to be dosimetrically superior to all these techniques, but their availability is limited at a few centers around the world.^(^ ^10^ ^)^

So far, only two investigators have utilized volumetric‐modulated arc therapy (VMAT) technique for planning APBI. This technique is a novel extension of conventional IMRT in which a three‐dimensional dose distribution may be delivered in a single gantry rotation.^(^ ^11^ ^)^ Qiu et al.^(^ ^12^ ^)^ reported the dosimetry of VMAT and 3D CRT in eight breast cancer patients previously treated with APBI. VMAT significantly reduced the volumes of ipsilateral breast irradiated to 5 Gy (from 70% to 59.6%, p=0.02), ipsilateral lung irradiated to 10 Gy (from 4.2% to 2%, p=0.03), and monitor units by 23% (p=0.02). However, the average maximum point dose to contralateral breast was increased from 175 cGy (4.5% of prescription dose) with 3D CRT to 256.3 cGy (6.7% of prescription dose) with RapidArc. Such dosimetric outcome would have excluded a patient from the RAPID trial.^(^ ^7^ ^)^ Shaitelman et al.^(^ ^13^ ^)^ used a medial and lateral continuous dynamic arc couch rotation (C‐ARC) for APBI treatment planning in 12 patients treated with 3D CRT. C‐ARC is similar to VMAT delivery in which the couch and not the gantry rotates in combination with a modulated beam aperture and dose rate. In their planning study, Shaitelman et al.^(^ ^13^ ^)^ limited the couch to rotate on an arc determined from the 3D CRT plan in order to avoid collision between couch, gantry, and the patient while the gantry was stationary. Compared with 3D CRT, C‐ARC significantly reduced ipsilateral lung volume receiving 5 Gy (7.8% versus 11.8%, p=0.001) and contralateral breast maximum dose (102.6 cGy versus 106.5 cGy, p=0.006). However, the impact of tumor location on the feasibility of APBI with VMAT was not addressed in these studies.

The Vancouver Island Centre (VIC) of the British Columbia Cancer Agency (BCCA) has been actively engaged in accruing patients for RAPID. Based on VIC statistics, approximately 25% of the patients who were eligible to receive APBI treatment did not participate in the RAPID trail because they failed to meet at least one of the dosimetric constraints imposed by the protocol. There are two main reasons why these patients had failed the planning constraints with 3D CRT. The first reason is the close proximity of the target volume to the contralateral breast (for lesions located in the upper‐inner part of the breast) which increased maximum point dose allowed for this OAR. The second is a large PTV (especially for lesions located in the outer part of the breast) which produced higher than the tolerance dose levels in the remaining ipsilateral breast.

Based on the improved dosimetry achieved by VMAT and C‐ARC techniques, we developed an APBI technique utilizing continuous couch and gantry dynamic arc therapy (CG‐Darc). In this dosimetric study, we have modeled the continuous dynamic couch rotation as a series of consecutive IMRT fields at 10° of couch interval combined with a simultaneous noncoplanar gantry rotation. We hypothesized that CG‐Darc would improve normal tissue dosimetry, as compared to 3D CRT and VMAT. This could potentially decrease the acute and late radiation‐induced toxicities, and would pave the way for expanding the availability of APBI to patients who currently fail dosimetric eligibility for such treatment.

## II. MATERIALS AND METHODS

### A. Patient selection

CT data sets from 35 breast cancer patients previously considered for APBI were used. Study patients were in two cohorts: Group A included 20 patients who were treated at our center with APBI under RAPID trial, and Group B consists of 15 patients who failed one or more dose constraints using 3D CRT and received WBRT because of trial ineligibility.

#### A.1 Group A

This group consisted of ten patients with right‐sided and ten with left‐sided tumors. To determine the potential impact of tumor location on dosimetric outcomes, two patients with tumors in each of the four breast quadrants (upper‐inner, upper‐outer, lower‐inner and lower‐outer) and an additional two with periareolar (central) tumors were selected for this study.

#### A.2 Group B

Ten of 15 patients who failed APBI dose constraints with 3D CRT had lumpectomy cavities located in the inner part of the breast (eight in upper‐inner) and contralateral breast maximum doses of more than 5%. Additionally, five patients with lesions in the outer breast were selected because they failed APBRT eligibility as a result of high ipsilateral breast dose (V50%>60% and/or V95%>35%).

### B. Definition of treatment volumes

Patients underwent standard CT simulation at 3 mm slice spacing, in the supine position on an angled breast board. The clinical target volume (CTV) was defined by circumferentially expanding the lumpectomy cavity (gross tumor volume) by 10 mm, but also subtracting the chest wall and the first 5 mm of breast tissue from the skin surface. The planning target volume (PTV) was created by adding a uniform margin of 10 mm around the CTV. For evaluation of target coverage, a dose evaluation volume (DEV) was defined as the PTV minus the chest wall, lung, and 5 mm of breast tissue from the skin surface. The PTV, which often extended outside the body contour, was used for field aperture generation. Accordingly, for VMAT and CG‐Darc optimization, in order to provide sufficient skin flash beyond the breast tissue, a 10 mm artificial bolus was added over the skin surface to accommodate variation due to setup uncertainties and patient breathing. The final dose calculation did not include the bolus.

The OARs considered in this study were: ipsilateral breast (extending from the suprasternal notch superiorly to 15 mm inferior to the inframammary fold), right and left lung, contralateral breast, heart, thyroid, and skin. The skin volume was defined as the superficial 5 mm of ipsilateral breast tissue.

### C. Treatment planning

The CT images were transferred to Eclipse treatment planning system (Varian Medical Systems, Palo Alto, CA) for radiotherapy treatment planning. Dose calculation was performed with anisotropic analytical algorithm (AAA) version 10.0.13 with 2.5 mm resolution.

All plans were generated using 6 MV photons from a Varian 21EX clinac (Varian Medical Systems) equipped with a millennium 120 multileaf collimator (MLC). For VMAT and CG‐Darc, the optimization goal was to reduce as low as possible the OAR dose without compromising the DEV homogeneity. The criteria used to choose the best plan were the minimum achievable ipsilateral lung V10% and contralateral breast maximum dose.

#### C.1 3D CRT planning

The 3D CRT plans were generated using a 4‐ or 5‐field, noncoplanar, photon‐only technique.^(^ ^1^ ^)^ Dynamic wedges were used to modulate the photon beams.

#### C.2 VMAT planning

The VMAT plans were generated using RapidArc (Varian Medical Systems) progressive resolution algorithm 10.0.13 (PR0 III). A single partial arc of 200° to 240° around the treated breast was used with an anterior avoidance sector of 90°–100° to prevent beam exit into intrathoracic OARs (heart, lung).^(^ ^12^ ^)^ The collimator angle range was between 10°–30°.

#### C.3 CG‐Darc planning

The continuous couch rotation was modeled by a series of consecutive fixed dynamic IMRT fields at 10° of couch interval. To avoid en face entrance beams, two tangential couch arcs were created with the same isocenter. For each couch arc, the gantry could simultaneously rotate in a noncoplanar arc for better avoidance of OARs. Using a trial and error method, we were able to determine the optimal couch arcs relative to gantry arcs. All the patients were planned using a medial couch arc of 60° and a lateral couch arc of 50°, as shown in Table 1. For each couch arc, the maximum noncoplanar gantry arc rotation was 20° but, depending on lesion location, the start and stop gantry angles were different. Because of the proximity of the contralateral breast and ipsilateral lung to the PTV, a wider gantry arc was not possible without increasing the dose to any one of these OARs. A gantry rotation of less than 5° was considered not clinically relevant and was set to zero.

**Table 1:** Couch angles used to generate the CG‐Darc plans. The angles are based on IEC (International Electrotechnical Commission, Geneva, Switzerland) convention.

*Lesion Location*	*Couch Angles, Medial Arc*	*Couch Angles, Lateral Arc*
Left	340° – 40°	10° – 320°
Right	20° – 320°	340° – 30°

### D. radiation dose prescription

The prescribed target (DEV) dose was 38.5 Gy in 10 fractions. The dose constraints and end points used for OAR evaluation were the ones specified in the RAPID protocol.^(^ ^1^ ^)^ These constraints were more stringent than the NSABP B‐39/RTOG0413 guidelines with regards to the radiation doses to ipsilateral breast, ipsilateral lung, and heart. The target coverage was also less restrictive than in the RAPID trail. The differences are outlined in Table 2.

**Table 2:** Dosimetric differences used for plan evaluation in RAPID and NSABP B‐39 clinical trials.

*OAR*	*RAPID Trial*	*NASBP B‐39/RTOG 0413*
Ipsilateral breast	V95%≤35 (up to 37%)	V100%≤35%
Ipsilateral lung	V10%≤20% (up to 23%) V30%≤10% (up to 13%)	V30% <15%
Heart		
Right‐sided	D5% <5%	V5% <5%
Left‐sided	5% D5% <15%	V5% <40%
Target (DEV)	V95%≥95%	V90%≥90% $D_{\max}$ ≤ 120%

### E. Plan comparison

The best CG‐Darc and RapidArc plans were compared with 3D CRT using the dose‐volume histograms (DVHs) and two‐sided paired t‐test statistical analysis. The results were considered significantly different if p≤0.05. For comparison, all the plans were normalized to DEV mean dose, as recommended in ICRU Report 83.^(^ ^14^ ^)^

For target volume evaluation, we used the volume of the DEV covered by 95% of dose, homogeneity index (HI),^(^ ^14^ ^)^ and Ian Paddick Conformity Index (IPCI),^(^ ^15^ ^)^ as defined below. The ideal plan would have a HI close to 0 and a CI close to 1:

(1) HI=(D2%−D98%)/Dmean

where, D2% and D98% were the near ‐ maximum and the near ‐ minimum absorbed dose to the DEV, expressed in Gy.

Ian Paddick Conformity Index was the fraction of the DEV enclosed by the reference dose (V95%) multiplied by the fraction of the total body volume included in the 95% isodose:

(2) IPCI=(DEV95%)/DEV*(DEV95%)/(V95%)

## III. RESULTS

### A. Group A

For the 20 patients treated with APBI, the mean PTV volume was 188 cc (range 108–317) with a mean ratio of PTV volume to ipsilateral breast volume of 0.15 (range 0.09–0.23).

**Figure 1:**
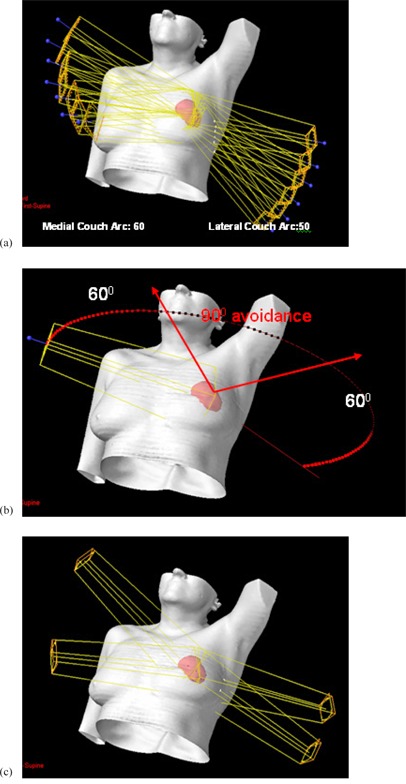
Typical beam arrangements for the three techniques to treat partial breast: (a) CG‐Darc, (b) RapidArc, and (c) 3D CRT.

**Figure 2:**
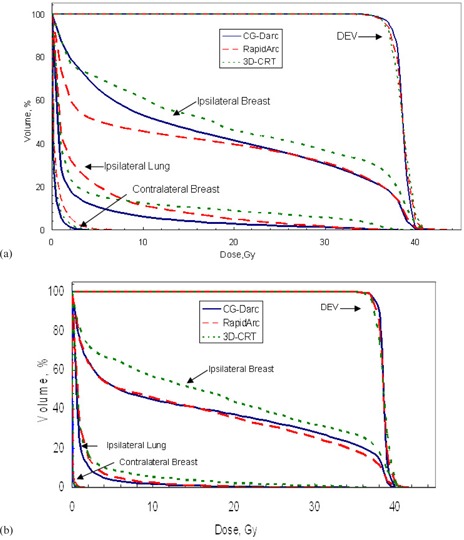
Representative DVHs of 2 Group A patients with lumpectomy cavities in the inner (a) and outer (b) quadrants. For the inner case (a), ipsilateral lung V10% was 23.7% and contralateral breast maximum dose was 6.9% with RapidArc, which exceeded the RAPID trail dose constraint. For the outer breast lesion (b), all the RAPID/NASBP‐B39 RTOG0413 trials dose constraints were met; however, CG‐Darc and RapidArc further reduced the ipsilateral breast overall dose as compared with 3D CRT.

A typical beam arrangement for CG‐Darc, RapidArc, and 3D CRT for a left‐sided centrally located tumor is shown in Figs. 1(a), 1(b), and 1(c), respectively. Figure 2 shows the DVH comparison between the three techniques for a representative a) upper‐inner and b) lower‐outer quadrant lesion location.

In all cases, CG‐Darc plans were dosimetrically superior to 3D CRT and RapidArc plans, as shown in Table 3 for central and inner lesions (12 cases) and in Table 4 for outer lesions (8 cases).

**Table 3:** Group A inner and central lesions location: DEV and OAR doses achieved with each technique. The average ± 1 standard deviation was over 12 patients (p‐value > 0.05 was not considered significant).

*Parameter (Objective)*	*3D CRT*	*CG‐Darc*	*RapidArc*	*p‐value CG‐Darc vs. 3D CRT*	*p‐value CG‐Darc vs. RapidArc*	*p‐value RapidArc vs. 3D CRT*
DEV						
HI (close to 0)	0.09±0.03	0.10±0.02	0.12±0.02	−	0.001	0.001
IPCI (close to 1)	0.40±0.07	0.55±0.08	0.59±0.09	<0.001	−	<0.001
V95%(>95%)	97.3±2.9	97.1±1.2	94.3±2.1	−	<0.001	<0.001
Max. Dose, %	105.2±2.2	105.0±1.5	106.9±1.1	−	0.008	−
Ipsilateral Breast						
V50% (<60%)	46.2±5.2	40.9±6.0	37.6±4.7	<0.001	0.002	<0.001
V95% (<35%)	21.1±4.4	15.7±3.6	13.8±2.8	<0.001	0.006	<0.001
V100% (<35%)	8.7±2.8	6.4±1.8	7.0±1.9	0.002	0.008	0.002
Mean dose, Gy	18.5±2.2	16.1±2.1	14.2±1.7	<0.001	<0.001	<0.001
Ipsilateral Lung						
V10% (<20%)	12.6±4.7	10.7±3.8	20.7± 8.8	0.04	<0.001	0.003
V30% (<10%)	6.7±3.1	4.5±2.1	8.3±3.4	0.004	<0.001	−
V20Gy (<3%)	4.5±1.9	2.4±1.2	3.6±1.9	0.001	0.002	−
Mean Dose, Gy	2.6±1.0	2.1±0.8	3.2±1.1	0.04	0.001	−
Contralateral Breast						
Max. Dose(<5%)	6.3±2.8	3.9±0.9	5.8±1.4	0.004	<0.001	−
Heart						
V5%,%	2.5±4.4	1.7±3.1	4.1± 8.9	−	−	−
Skin						
Mean Dose, Gy	15.4±1.6	12.7±2.0	10.7±2.0	<0.001	<0.001	<0.001
D2%,Gy	37.4±0.6	36.2±1.8	34.6±1.4	0.03	0.002	<0.001

**Table 4:** Group A outer lesions location: DEV and OAR doses achieved with each technique. The average ± 1 standard deviation was over 8 patients (p‐value > 0.05 was not considered significant).

*Parameter (Objective)*	*3D CRT*	*CG‐Darc*	*RapidArc*	*p‐value CG‐Darc vs. 3D CRT*	*p‐value CG‐Darc vs. RapidArc*	*p‐value RapidArc vs. 3D CRT*
DEV						
HI (close to 0)	0.08±0.01	0.08±0.01	0.09±0.01	−	−	−
IPCI (close to 1)	0.42±0.09	0.55±0.13	0.63±0.07	0.002	0.02	<0.001
V95%(>95%)	99.2±1.1	99.4±0.42	98.2±0.6	−	0.001	−
Max. Dose, %	105.2±1.8	105.5±1.2	107.5±1.1	−	0.01	0.004
Ipsilateral Breast						
V50% (<60%)	45.1±8.0	37.4±7.2	36.4±6.0	0.003	−	<0.001
V95% (<35%)	24.1±4.3	19.2±4.2	16.2±2.6	<0.001	0.02	<0.001
V100% (<35%)	11.6±3.9	7.8±2.8	6.8±1.4	0.001	−	0.004
Mean dose, Gy	17.6±2.9	15.1±2.7	14.4±2.2	0.001	−	<0.001
Ipsilateral Lung						
V10% (<20%)	9.7±6.0	6.1±4.3	9.4±5.3	0.006	0.004	−
V30% (<10%)	4.4±3.2	1.9±1.7	2.5±1.8	0.007	−	−
V20Gy (<3%)	2.3±2.0	1.0±1.0	0.8±0.7	−	−	0.04
Mean Dose, Gy	2.0±1.1	1.3±0.7	1.6±0.7	0.006	0.002	−
Contralateral Breast						
Max. Dose(<5%)	3.8±3.7	2.5±1.0	4.0±1.4	−	0.008	−
Heart						
V5%, %	0.9±1.4	0.8±1.4	2.4±3.0	−	0.04	−
Skin						
Mean Dose, Gy	14.2±0.7	12.1±1.5	11.1±0.9	0.002	0.01	<0.001
D2%,Gy	37.4±1.2	37.3±1.6	35.7±0.8	0.01	0.03	0.02

Compared with 3D CRT, CG‐Darc significantly reduced the ipsilateral lung volume receiving 10% and 30% of the prescribed dose, ipsilateral breast volume receiving 50% and 95% of the prescribed dose, and skin mean and maximum dose (p≤0.04). For the most difficult cases (central and inner lesions), CG‐Darc was also able to significantly reduce the contralateral breast maximum dose (3.9% vs. 6.3%, p=0.004) while maintaining coverage with improved conformity of DEV (p=0.001).

RapidArc plans were dosimetrically comparable to the 3D CRT plans for lesions located in the outer part of the breast, as shown in Table 4. For the 12 patients with lesions located in the central and inner part of the breast, the ipsilateral lung volume receiving 10% of the dose was on average 20.7%, while the contralateral breast maximum dose was 5.8%. However, in half of these cases, RapidArc was not able to achieve the desired dose constraints for these critical structures. For this subgroup of patients, the average ipsilateral lung V10% was 27.5% (range: 22.4%−40.3%), while the average contralateral breast maximum dose was 6.9% (range: 6.1%−7.8%) of the prescribed dose. In these cases, the coverage and homogeneity of DEV was also suboptimal. As shown in Table 3, the CG‐Darc technique was significantly better than RapidArc at reducing the dose to ipsilateral lung and contralateral breast (all p≤0.002) while simultaneously improving homogeneity and coverage of DEV (p≤0.001).

The main feature of RapidArc was a further reduction in the overall dose to the ipsilateral breast and ipsilateral skin dose, as compared with CG‐Darc (p≤0.03). This reduction was more pronounced for lumpectomy cavities located in the central and inner part of the breast (p≤0.006).

Averaged on 20 patients, the treatment time was 1.6 minutes with RapidArc and 6 minutes with 3D CRT. The number of MU was 550 with RapidArc versus 514 with 3D CRT.

### B. Group B

**Figure 3:**
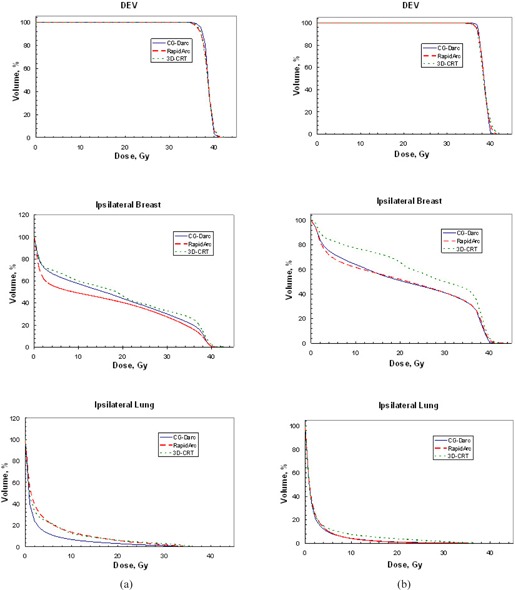
Group B mean DVHs for ipsilateral lung, ipsilateral breast, and DEV. Column (a) averaged over 10 patients with inner/central lesions the ipsilateral lung dose was significantly reduced with CG‐Darc. Column (b) averaged over 5 patients with outer lesions the ipsilateral breast dose was significantly reduced with CG‐Darc and RapidArc.

**Figure 4:**
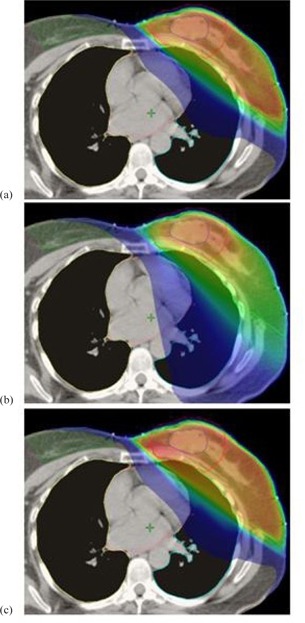
Example of a dose distribution on a CT axial slice down to 5% level for a Group B upper‐inner case: (a) CG‐Darc, (b) RapidArc, and (c) 3D CRT. The low‐dose splash to normal tissue is the same for CG‐Darc and 3D CRT, but more for RapidArc.

For the 10 patients with lesions in the inner breast, the mean PTV volume was 189 cc (range 102–311) with a ratio of PTV volume to ipsilateral breast volume of 0.15 (0.07–0.26). For the five patients with lesions in the outer breast, the mean PTV volume was 434 cc (range 260–522) with a mean ratio of PTV volume to ipsilateral breast volume of 0.25 (0.21–0.30). The best CG‐Darc plans were generated with the same template as before for the medial and lateral couch arcs (Table 2). The summarized results for the three techniques are presented in Table 5 for the inner cases and Table 6 for outer cases. Mean DVH comparison between the three techniques, for ipsilateral breast, ipsilateral lung, and DEV is also shown in Fig. 3(a) for inner lesions and 3(b) for outer lesions. Color wash dose distribution down to 5% dose level is displayed in Fig. 4 for a patient with an upper‐inner lesion.

**Table 5:** Group B inner and central lesions location: DEV and OAR doses achieved with each technique. The average ± 1 standard deviation was over 10 patients that failed one or more RAPID trail dose constraints (p‐value > 0.05 was not considered significant).

*Parameter (Objective)*	*3D CRT*	*CG‐Darc*	*RapidArc*	*p‐value CG‐Darc vs. 3D CRT*	*p‐value CG‐Darc vs. RapidArc*	*p‐value RapidArc vs. 3D CRT*
DEV						
HI (close to 0)	0.11±0.04	0.09±0.02	0.13±0.02	−	<0.001	−
IPCI (close to 1)	0.39±0.11	0.49±0.08	0.58±0.10	0.002	0.005	<0.001
V95%(>95%)	95.4±4.1	97.0±1.7	93.5±2.3	−	0.001	−
Max. Dose, %	106.8±2.4	105.5±1.6	107.5±1.5	−	0.03	−
Ipsilateral Breast						
V50% (<60%)	48.3±15.3	45.2±10.7	41.1±12.2	−	0.02	0.01
V95% (<35%)	22.9±11.2	18.0±8.2	14.3±6.5	0.01	0.01	0.001
V100% (<35%)	10.6±5.0	7.9±3.7	6.7±3.4	0.02	−	0.006
Mean dose, Gy	18.7±5.2	17.5±4.2	15.4±4.2	−	0.001	<0.001
Ipsilateral Lung						
V10% (<20%)	25.2±19.1	15.1±5.8	27.3±11.0	−	<0.001	−
V30% (<10%)	10.7±5.9	6.0±2.6	12.0±5.7	0.02	0.001	−
V20Gy (<3%)	6.0±3.5	3.0±1.3	5.9±3.6	0.01	0.01	−
Mean Dose, Gy	4.3±2.2	2.4±1.2	4.3±1.7	0.02	<0.001	−
Contralateral Breast						
Max. Dose(<5%)	9.2±3.7	4.3±0.7	6.3±1.5	0.003	<0.001	0.02
Heart						
V5%, %	12.9±29.2	3.1±5.7	5.0±8.6	−	0.04	−
Skin						
Mean Dose, Gy	13.8±5.0	12.1±3.9	9.7±4.6	0.03	0.01	<0.001
D2%,Gy	36.7±2.0	35.6±1.8	34.9±2.3	0.01	−	0.005

**Table 6:** Group B outer lesions location: DEV and OAR doses achieved with each technique. The average ± 1 standard deviation was over 5 patients that failed one or more RAPID trail dose constraints (p‐value > 0.05 was not considered significant).

*Parameter (Objective)*	*3D CRT*	*CG‐Darc*	*RapidArc*	*p‐value CG‐Darc vs. 3D CRT*	*p‐value CG‐Darc vs. RapidArc*	*p‐value RapidArc vs. 3D CRT*
DEV						
HI (close to 0)	0.12±0.03	0.08±0.004	0.11±0.02	0.04	0.04	−
IPCI (close to 1)	0.44±0.06	0.65±0.09	0.62±0.06	0.001	−	0.009
V95%(>95%)	96.4±2.8	98.9±0.6	96.7±2.3	−	−	−
Max. Dose, %	108.0±2.0	105.9±0.3	109.4±1.4	−	0.002	−
Ipsilateral Breast						
V50% (<60%)	67.5±4.7	51.6±5.0	52.6±3.1	0.02	−	0.001
V95% (<35%)	38.3±3.4	28.9±2.1	28.9±5.4	0.006	−	0.01
V100% (<35%)	17.8±2.1	12.2±1.0	13.1±3.7	0.01	−	0.02
Mean dose, Gy	25.2±1.5	20.8±1.3	20.6±1.4	0.02	−	0.001
Ipsilateral Lung						
V10% (<20%)	15.2±6.7	12.4±2.4	13.9±4.1	−	−	−
V30% (<10%)	6.7±3.7	3.3±0.8	3.1±1.8	−	−	−
V20Gy (<3%)	3.7±1.6	1.0±0.6	0.9±0.6	0.04	−	−
Mean Dose, Gy	2.9±0.9	2.1±0.3	2.2±0.4	−	−	−
Contralateral Breast						
Max. Dose(<5%)	7.7±10.8	2.5±1.5	4.1±0.9	−	−	−
Heart						
V5%, %	2.2±3.1	2.8±4.3	5.8±9.4	−	−	−
Skin						
Mean Dose, Gy	20.1±2.6	14.9±1.8	14.9±2.7	0.004	−	0.001
D2%, Gy	38.4±0.8	35.8±1.0	36.1±1.5	<0.001	−	0.002

For inner and central lesions, CG‐Darc achieved the lowest maximum point dose to contralateral breast (4.3%±0.7%) and reduced ipsilateral lung dose, with an average V10% of 15.1%±5.8% while meeting all the other dose restrictions. On average, RapidArc plans were not able to reduce the contralateral breast maximum dose below 5% (6.3%±1.5%) and V10% for ipsilateral lung below 20% (27.3%±11.0%). The volume of DEV receiving 95% of the prescription dose was also inferior with RapidArc compared to CG‐Darc (93.5%±2.3% versus 97.0%±1.7%, p=0.001).

For outer lesions location with CG ‐Darc technique, the ipsilateral breast dose was significantly reduced as compared with 3D CRT and comparable with RapidArc dose (V50%: 51.6% vs. 67.5%, p=0.02 and 52.6%, respectively).

## IV. DISCUSSION

This present study reported the dosimetry of a new technique that utilizes dynamic arc rotation of both couch and gantry (CG‐Darc) and compared it against RapidArc and 3D CRT. The dynamic couch arc rotation was simulated by utilizing a series of sequential IMRT fields simultaneous with noncoplanar gantry rotation. The modulated couch and gantry motion of CG‐Darc provides more degrees of freedom in radiation delivery of APBI, therefore avoiding beams that are directed towards the critical structures of the chest. This is of particular importance in patients with challenging anatomy. From our experience, patients with large or central lesions have difficulty achieving ipsilateral breast V50% less than 60% and ipsilateral lung V10% less than 20%. An additional challenge for inner breast lesions has been to achieve a contralateral breast maximum dose of <5% of dose prescription.

In this study, a maximum contralateral breast point dose of 5% was used instead of the recommended 3% by the RAPID protocol.^(^ ^1^ ^)^ This is because all the plans were calculated utilizing the AAA algorithm which has been reported to overestimate the contralateral breast dose by 2%–3% compared to Monte Carlo algorithm.^(^ ^16^ ^)^ This difference is mostly due to the head scatter and leakage modeling within AAA, and led to the recommendation of relaxing the contralateral breast constraint to a maximum point dose of 5% of the prescribed dose.

The results of this study confirmed the hypothesis that CG‐Darc had superior target and OAR dosimetry compared to RapidArc and 3D CRT. Although 3D CRT achieved doses within the prescribed dose constraints for Group A patients, further dose reduction may limit the risk of long‐term complications and secondary malignancies. Such an example was provided in the report by Jagsi et al.^(^ ^17^ ^)^ In their study, APBI was delivered using IMRT with active breathing control. Unfortunately, the study was prematurely closed because of unacceptable chronic skin toxicity, which was surprisingly found to be associated with ipsilateral breast V50% above 40% and V100% above 20%. This was well below the RAPID and NSABP B‐39 recommended V50% of 60% and V100% of 35%, respectively. Hapel at el.^(^ ^5^ ^)^ found that ipsilateral breast V5% and V20% (related with V80% and V100% in 3D CRT for APBI) were predictive of worse cosmesis results. Longer clinical follow‐up in larger number of patients will determine the true incidence of APBI chronic toxicities.

In Group B of patients that failed dose constraints of RAPID trail, CG‐Darc improved dosimetry in all patients. This would have enabled them to receive APBI if this technique was available in routine clinical practice. Failure to achieve the lung dose constraints and excessively high dose to contralateral breast with 3D CRT were the primary reasons for APBI exclusion of patients with inner/central lesions. Increased low dose spillage to the ipsilateral lung and contralateral breast is also noted with RapidArc in this subgroup of patients. Specifically, both 3D CRT and RapidArc resulted in lung V10% in excess of 20% and V20 Gy of 3% that have been reported by Recht et al.^(^ ^6^ ^)^ to increase the risk of pneumonitis by 17% and 20%, respectively. As shown in Table 5, the dose to the contralateral breast was also prohibitive for APBI with both 3D CRT and RapidArc.

The increase in lung, heart, and contralateral breast volume receiving low doses of radiation (V5% of dose prescription) by RapidArc is well illustrated in Fig. 4 for an upper inner lesion case. Review of data from a larger cohort of VIC breast cancer patients with inner/central lumpectomy cavities demonstrated failure of all three APBRT techniques to spare the contralateral breast in tumors located very close to midline. As illustrated in Fig. 4, a distance of less than 5 cm between DEV and contralateral breast in any CT axial slice increased the scatter dose and resulted in higher‐than‐recommended contralateral breast maximum point dose. In addition, patients with PTV to ipsilateral breast ratio higher than 30% also failed all three techniques, as it was impossible to satisfy the ipsilateral breast radiation dose constraints. Such patients had traditionally been excluded from the APBI clinical trials.

For patients with outer breast lesions, CG‐Darc and RapidArc both significantly reduced ipsilateral breast V50% by an absolute mean value of 8% in Group A and 15% in Group B, as compared with 3D CRT technique. As a consequence, the skin mean and maximum dose was also significantly reduced, as shown in Tables 4 and 6. The skin sparing achieved with these two techniques would be beneficial in reducing toxicity for all patients and especially for large‐breasted women.

As a corollary, this study has shown that RapidArc may only be indicated in patients that have lesions located in the outer part of the breast. The advantages of using RapidArc against 3D CRT were the reduced overall ipsilateral breast dose, skin dose, and treatment time.

Skin flash has to be incorporated into any VMAT treatment for breast. In this study, for RapidArc and CG‐Darc planning, an artificial bolus was added to the optimization process to account for potential movement of the breast during treatment, but was not included in the final dose calculation. Another approach to account for intrafraction respiratory motion and possible residual changes in the target during radiotherapy of the whole breast for VMAT delivery is to extend the CT dataset (only in the region of the breast) and PTV outside the body by 1 cm when optimizing the plans. This skin flash method was successfully implemented by Nicolini et al.,^(^ ^18^ ^)^ and showed improved target coverage without under‐dosage during treatment.

This study demonstrated the feasibility and dosimetric superiority of CG‐Darc compared to RapidArc and 3D CRT. If deployed in routine clinical practice, it will broaden patient eligibility for APBI and has the potential of further shifting the radiotherapy practice paradigm in breast cancer. For simplicity, in this study the two couch arcs have the same isocenter. There is no collision between the couch and gantry for the medial arc. For the lateral arc, in order to ensure gantry rotation clearance, the couch may need to be translated. For clinical implementation, the rotation and translation of the couch during treatment has to be incorporated into the RapidArc algorithm. To make this technique efficient, the couch motions have to be linked to the existing dynamic gantry arc rotation and variable dose rate. This is not trivial, but can be done as demonstrated by a group from Memorial Sloan Kettering Cancer Centre. Recently, this group published an algorithm called tra‐VMAT^(^ ^19^ ^)^ that integrates couch and collimator trajectories into RapidArc. This technique is deliverable on both Varian TrueBEAM system and traditional accelerators. Tra‐VMAT resulted in better target dose coverage/homogeneity and OAR sparing compared to standard VMAT with only gantry rotation, in 10 patients with brain tumors. We agree with the above researchers that, because of the complexity of such treatment, the vendor should develop a new patient safeguard system to prevent any collisions from happening, and additional physics quality assurance programs should be implemented before clinical use. Also, couch motion during treatment may potentially affect patient positioning accuracy and needs to be carefully assessed in future study. This additional work would be justified if a couch‐gantry dynamic rotation proves to be advantageous in other tumor sites.

## V. CONCLUSIONS

This planning study provided proof of principle that simultaneous dynamic couch and gantry rotation is feasible for APBI. It minimized OAR radiation dose exposure without compromising target coverage in all patients. It is the only technique that generates favorable dosimetry in patients with inner/central breast lesions currently failing dose constraints. As a result, CG ‐Darc has the potential to improve the therapeutic window of APBI by expanding its applications to groups of patients currently ineligible for such treatment. A modification to the RapidArc algorithm is needed to enable the use of CG‐ Darc in routine clinical practice.

## ACKNOWLEDGMENTS

This study was supported in part by a research grant to the BC Cancer Agency from Varian Medical Systems, Inc and in part by the BC Cancer Foundation.
